# 

**Published:** 1851-01-01

**Authors:** 


### THE JOURNAL

OF

### PSYCHOLOGICAL MEDICINE

AND

### MENTAL PATHOLOGY.

EDITED BY

FORBES WINSLOW, M.D.

S.G.O.L.

Surgeon Gen'l's Office
LIBRARY
22048
Washington, D.C.

VOL. IV.

LONDON:

JOHN CHURCHILL, PRINCES STREET, SOHO.

MDCCCLI.

LSGO

W
1
JO 857 N

### To Correspondents.

We have again to apologize to our correspondents for an unavoidable postponement of much valuable matter. By giving an extra sheet with this Number, we thought we should be able to notice all the articles, books, and pamphlets forwarded to us. We have, however, been disappointed. In the April number we hope to make ample amends for all "sins of omission." We purpose devoting a considerable part of the next number of the Journal to an analysis of the books, pamphlets, and papers which our kind friends have sent to us, and to the publication of much miscellaneous matter relating to judicial insanity. The American and German Psychological Journal will also be fully analysed, and the works of Mr. Grantham and Dr. Burnett reviewed at length. No. 2 of the Editor's Portfolio will also appear in our April number.

